# A systematic review of the Da Vinci® Single-Port system (DVSP) in the context of colorectal surgery

**DOI:** 10.1007/s00384-025-04878-x

**Published:** 2025-04-02

**Authors:** Francesco Brucchi, Isacco Montroni, Roberto Cirocchi, Giovanni Taffurelli, Marco Vitellaro, Gianluca Mascianà, Giovanni Battista Levi Sandri, Gianlorenzo Dionigi, Sara Lauricella

**Affiliations:** 1 University of Milan, 20122 Milan, Italy; 2 Colorectal Surgery Unit, Fondazione IRCCS Istituto Nazionale Dei Tumori, Via Venezian 1, 20133 Milan, Italy; 3 Digestive and Emergency Surgery Unit, S.Maria Hospital Trust, 05100 Terni, Italy; 4 Department of Medicine and Surgery, University of Perugia, Piazza Università 1, 06123 Perugia, PG Italy; 5 Colorectal Surgery Clinical and Research Unit, Fondazione Policlinico Universitario Campus Bio-Medico Di Roma, University Campus Bio-Medico, 00128 Rome, Italy; 6 Surgical Department, Ospedale “Leopoldo Delfino Parodi”, Asl Roma 5, Colleferro (RM), Rome, Italy; 7 Division of Surgery, Istituto Auxologico Italiano IRCCS (Istituto Di Ricovero E Cura a Carattere Scientifico), Via Giuseppe Mercalli, 30, 20122 Milan, Italy; 8 Department of Pathophysiology and Transplantation, University of Milan, Milan, Italy

**Keywords:** Robotic surgery, Colorectal surgery, Minimally invasive surgery, Colorectal cancer, Robotic surgical systems, Da Vinci, Da Vinci SP, Single-port

## Abstract

**Purpose:**

The purpose of this study is to review the application of the da Vinci® Single-Port system (DVSP) in colorectal surgery, with a view to assessing its safety and feasibility, and investigating its clinical and oncological outcomes.

**Methods:**

A comprehensive search of the scientific literature was conducted across three major databases (PubMed, Web of Science, and Cochrane) up to November 2024. The study was registered in PROSPERO (CRD42024612762) and conducted in accordance with the Preferred Reporting Items for Systematic Reviews and Meta-Analyses (PRISMA) guidelines. Included studies pertained to the utilisation of DVSP in the domain of colorectal surgery.

**Results:**

Eleven articles were included in the final analysis. No randomized controlled trials were identified. A total of 396 patients (199 men, 197 women) underwent robotic colorectal surgery using the DVSP. Surgical resections were indicated for benign pathology in 56 patients, colon carcinoma in 194 cases, and rectal carcinoma in 146 cases. The median incision length for Uniport placement was 4 cm. The median docking time was 5.96 min (IQR, 9.33 min), and the median console time was 105 min (IQR, 62.51 min). The mean operative time was 186.3 min (IQR, 77.65 min). Intraoperative complications were rare, with only two cases reported (0.47%). Postoperative complications occurred in 12.47% of patients, with ileus being the most common. No patients were readmitted for complications within 30 days. Short-term oncological outcomes seemed promising, with a median of 24.59 lymph nodes retrieved. There were no reported deaths within 30 days. The median follow up time was 11.4 months (IQR, 11.76 months).

**Conclusion:**

This study shows that the use of DVSP in colorectal surgery is both feasible and safe. Short-term clinical and oncological outcomes seem promising. However, longer follow-up data and larger patient cohorts are needed to fully assess the long-term efficacy of this novel technique.

**Prospero registry:**

Registration number CRD42024612762.

**Supplementary Information:**

The online version contains supplementary material available at 10.1007/s00384-025-04878-x.

## Introduction

Colorectal cancer (CRC) is the third most commonly diagnosed malignancy and the second leading cause of cancer-related death on a global scale [1]. The incidence of CRC exhibits geographical and ethnic variations and is influenced by age, with a notable increase in cases among individuals younger than 50 years over the past few decades [2]. Laparoscopy has progressively replaced traditional open abdominal surgery for the treatment CRC, offering several advantages, including shorter hospital stays, reduced postoperative pain, quicker return of bowel function, and lower overall morbidity[3–5].

However, the laparoscopic approach is subject to inherent technical limitations that can compromise its efficacy and safety. These limitations include spatial constraints, two-dimensional vision, restricted degrees of freedom, disrupted eye-hand coordination, and instrument conflicts. Laparoscopic instruments are straight and rigid, requiring surgeons to perform intricate, camera-dependent movements within the confined space of the bony pelvis [6–10].

The advent of surgical robots signifies a substantial advancement in minimally invasive surgery, characterized by substantial technological improvements that enhance surgical precision and patient outcomes. Robotic surgical systems offer three-dimensional, high-definition visualization, facilitating superior depth perception and magnification compared to traditional laparoscopy. This enhanced visualization is crucial for performing intricate procedures in confined anatomical spaces, such as the pelvis in rectal surgery, which may be challenging with traditional laparoscopic techniques. Furthermore, robotic systems offer increased degrees of freedom, enhanced precision in surgical manoeuvres, and improved control and dexterity. Ergonomics is another significant advantage of robotic surgery, as surgeons operate from a console, reducing the physical strain and fatigue associated with traditional laparoscopic surgery [11, 12]. However, it is imperative to consider the drawbacks, including increased operative times, substantial costs, and the necessity for specialized training [13].

The advent of the robotic Single Port system represents a recent development in minimally invasive surgery, integrating the benefits of robotic surgery, such as precision and control, with the cosmetic and recovery benefits of single-incision laparoscopic surgery (SILS). The SP system allows for the insertion of multiple robotic instruments through a single port, typically positioned in the umbilicus or a suprapubic location, thereby minimizing the number of incisions required. This stands in contrast with traditional multiport robotic or laparoscopic surgeries, which require multiple incisions. The da Vinci® Single-Port system (Intuitive Surgical, Sunnyvale, CA, USA) is a prominent example of this technology. To date, its use has been predominantly in urological and gynaecological procedures [14–16]. The present study aims to review the data on the application of the da Vinci® Single-Port system (DVSP) in colorectal surgery, assess the feasibility, and investigate its clinical outcomes.

## Materials and methods

A comprehensive online systematic search was conducted using PubMed, Web of Science, and Cochrane databases for eligible articles until November 11, 2024. A combination of keywords was used in the search: “robotic,” “colorectal surgery,” “minimally invasive surgery,” “colorectal cancer,” “robotic surgery,” “robotic surgical systems,” “Da Vinci,” “Da Vinci SP,” and “single-port.” This systematic review was reported in accordance with the PRISMA (Preferred Reporting Items for Systematic Reviews and Meta-Analyses) 2020 Statement [17] and was pre-registered with PROSPERO (registration number: CRD42024612762). The AMSTAR (A Measurement Tool to Assess Systematic Reviews) checklist was included in the Supplementary Material [18]. The research encompassed original scientific manuscripts, comparative studies, review articles, meta-analyses, and case series. Exclusion criteria included the following: case reports, articles in non-English languages, articles unrelated to the review topic, studies for which complete articles were unavailable, and patients receiving different surgical procedures than colorectal. No restrictions were applied regarding patient age. Duplicates were excluded, including both articles replicated across multiple databases and studies analyzing the same patient cohort, to prevent data overlap.

The selection process was conducted by SL and FB, who independently reviewed the titles and abstracts of each article and subsequently assessed the full-text articles against the predetermined eligibility criteria. Disagreements were resolved through discussion or, if unresolved, through arbitration with a third reviewer (RC).

## Research database and outcomes

For each study, the following data were extracted: year of publication, country, study design, number of patients enrolled, demographic and clinical characteristics (sex, age, body mass index (BMI), American Society of Anaesthesiologists (ASA) classification, previous abdominal surgical history, indication for surgery (benign or malignant), tumor location, distance of the tumor from the anal verge, preoperative clinical staging, preoperative treatment, and type of operation performed), as well as intraoperative outcomes (type of platform used, robotic single-port placement, total incision length in centimetres, operative time for each step, including total operative time, docking time, and total console time), need for additional ports, incision site for specimen retrieval, conversion to laparoscopic or open approaches, estimated blood loss, use of drains, diverting stoma, and any complications were also investigated.

Postoperative outcomes, including hospital stay, time to flatus, time to consuming a soft diet, complications, readmissions, mortality within 30 days, and oncological outcomes, were also investigated. Postoperative morbidities were classified according to the Clavien–Dindo classification system [19]. The quality of non-randomized controlled trials (NRCTs) was assessed using the Newcastle–Ottawa Scale (NOS), with scores ranging from 5 to 6 stars, indicating moderate quality [20]. Two researchers independently assessed each study using the Review Manager tool (RevMan, version 5.4, The Cochrane Collaboration), focusing on five key domains: bias arising from the randomization process, deviations from intended interventions, missing outcome data, measurement of the outcome, and selection of the reported result. Discrepancies were resolved through consultation with a third-party expert.

## Results

### Assessment of quality of the included studies

**Fig. 1:**
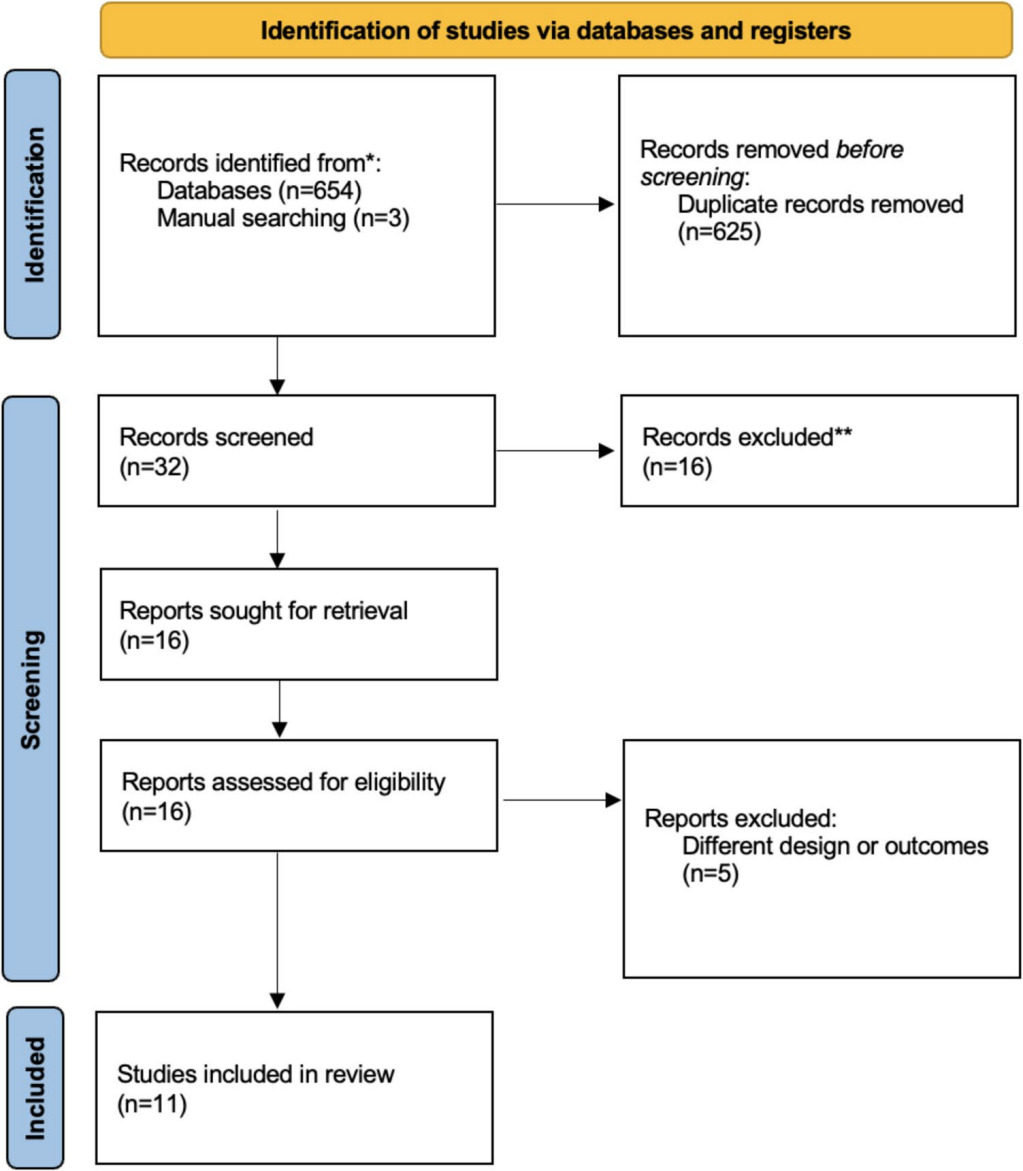
Flowchart of study screening according to PRISMA guidelines

The comprehensive literature search yielded 657 publications, which were then subjected to a rigorous quality assessment. Of these, 654 were retrieved from electronic databases and three were identified through manual searching (Fig. 1). Following the removal of duplicates and screening the title and abstract, 32 full-text articles were selected for eligibility assessment. Eleven articles [21–31] were included in the final analysis (nine retrospective [22–26, 28–30] and two prospective studies [21, 27]). No randomized controlled trials were identified. The characteristics of the included studies are outlined in Table 1, including the authors, year of publication, country, study design, sample size, and indication for the procedure. A thorough risk of bias assessment is provided in the supplementary materials (see Fig. 1s).

**Table 1:** Studies included in the review

Author	Year	Enrollment	Country	Study design	Total number of patients	Indication for surgery (benign)	Indication for surgery (colon cancer)	Indication for surgery (rectal cancer)
Song et al. [21]	2021	July–September 2020	Korea	Prospective	5	0	5	0
Ha Lim et al. [27]	2022	January 2019–December 2020	Korea	Prospective	41	0	41	0
Piozzi et al [22]	2022	November 2020 to December 2021	Korea	Retrospective	13	1	5	7
Alshalawi et al. [23]	2023	November 2021–February 2022	Saudi Arabia, Korea	Retrospective	15	0	0	15
Hye Jeong et al. [29]	2023	Whole 2020	Korea	Retrospective	13	0	0	13
Jin Kim et al. [32]	2023	July 2020–June 2022	Korea	Retrospective	42	0	0	42
Jung et al. [26]	2023	November 2020–December 2022	South Korea	Retrospective	53	0	53	0
H. S. Kim et al. [31]	2023	March 2019–September 2021	South Korea	Retrospective	50	11	39	0
Marks et al. [25]	2023	October 2018–August 2021	USA	Retrospective	115	44	12	59
Jung Cho et al. [28]	2024	May 2023–December 2023	Korea	Retrospective	10	0	9	1
Suk Choi et al.	2024	April–October 2019	Korea	Retrospective	39	0	30	9

### Landmark papers

The article published by Song et al. [21] in 2021 represents one of the first case series available on the use of DVSP in colorectal surgery, with the exception of individual case reports. The key study within our systematic review with the largest case series was published by Marks et al. in 2023 and includes 93 colorectal resections performed using DVSP [25]. There are no temporal gaps in the analyzed publications, which span from 2021 to 2024. All the studies included in the analysis originate from Korea [21, 22, 24, 26–31], except for Marks et al.’s study [25], which was conducted in the USA and Alshalawi et al.’s study [23] conducted in Saudi Arabia. None of the analyzed studies included a cost analysis of this system, leaving its economic impact and cost-effectiveness unexplored.

### Patient demographic characteristics

The baseline characteristics of patients from the included studies are shown in Table 2. A total of 396 patients (199 men, 197 women) underwent robotic colorectal surgeries using the DVSP system.

**Table 2:** Patients demographics

Author	ASA score, *n*	Age, mean ± SD	BMI, mean ± SD	Sex ratio, *n* (M/F)	Prior abdominal surgery, *n*
Song et al. [21]	I = 4 II = 1	69 (58–77)*	24 (23.4–26.8)*	4/1	1
Ha Lim et al. [27]	I = 9 II = 30 III = 2	60.80 ± 11.17	23.80 ± 3.16	20/21	10
Piozzi et al. [22]	II = 12 III = 1	56.6 ± 10.4	22.87 ± 3.63	9/4	NR
Alshalawi et al. [23]	I = 3 II = 11 III = 1	61 (49–80)*	22.21 (18.4–29.4)*	8/7	0
Hye Jeong et al. [29]	I = 5 II = 7 III = 1	53.2 ± 12.9	23.4 ± 3.5	7/6	NR
Jin Kim et al. [32]	I = 6 II = 29 III = 7	62.5 (9.8)*	24.4 (2.7)*	28/14	NR
Jung et al. [26]	I-II = 50 III-IV = 3	60.4 (9.7)*	23.3 (3.2)*	34/19	NR
H. S. Kim et al. [31]	I = 9 II = 40 III = 1	59.0 (52.5–63.0)*	24 (21.0–26.0)*	26 (52)/24 (48)	NR
Marks et al. [25]	2 (1–3)*	59.74 (11.92)*	27.52 (6.31)*	56 (42)/77 (58)	59 (44.36)
Jung Cho et al. [28]	I = 1 II = 4 III = 5	62.9 ± 9.6	22.87 ± 2.5	4/6	NR
Suk Choi et al. [39]	I = 4 II = 32 III = 3	61.28 ± 13.03	23.79 ± 2.86	23/16	NR

^*^Median (range), *ASA* American Society of Anesthesiologists, *BMI* body mass index, *NR* not reported, *SD* standard deviation, *M* male, *F* female

The median age of the patients was 60.6 (interquartile range (IQR) = 4.06), and the median BMI was 23.6 kg/m^2^ (interquartile range (IQR) = 0.65). The most prevalent American Society of Anesthesiologists (ASA) classification was II. Surgical resection was performed for benign pathology in 56 patients, colon carcinoma in 194 cases, and rectal carcinoma in 146 cases. The surgical procedures included 118 (34.3%) rectal anterior resections with total mesorectal excision (TME), 123 (32.4%) right colectomies, 30 (8.6%) left colectomies, 30 (7.2%) transanal transabdominal radical proctosigmoidectomy (TATA), 27 (6.5%) intersphincteric resections, 15 (3.6%) sigmoid resections, 12 (2.9%) total proctectomy with ileoanal anastomosis, 6 (1.4%) abdominoperineal resections, 2 (0.5%) rectopexy, and 1 (0.2%) Hartmann reversal (see Table 3 for details). The median follow-up time was 11.4 months (IQR, 11.76 months).

**Table 3:** Oncologic details and technical aspects

Author	Distance from the a.v. (cm), median	Clinical T stage	Clinical N stage	Neoadjuvant chemoradiation, *n* (%)	RC (CME)	LC (CME)	Sigmoid resection (CME)	RAR + TME	TATA	Intersphincteric resection
Song et al. [21]	NR	cT1(0), cT2(1), cT3(3), cT4 (1)	cN0(1), cN + (4)	2 (40)	5	0	0	0	0	0
Ha Lim et al. [27]	NR	NR	NR	0	41	0	0	0	0	0
Piozzi et al. [22]	2.79 ± 0.45*	NR	NR	7 (53.8)	5	0	0	0	0	7
Alshalawi et al. [23]	10 (2–15)	NR	NR	5 (33.3)	0	0	0	12	0	3
Hye Jeong et al. [29]	4.0 (3.0–8.0)	cT1 (2), cT2 (4), cT3 (7), cT4 (0)	cN0(4), cN + (9)	5 (38.4)	0	0	0	8	0	5
Jin Kim et al. [32]	5.9 ± 2.1 cm*	cT1/2 (20), cT3 (22)	NR	26 (61.9)	0	0	0	30	0	12
Jung et al. [26]	NR	cT0-2 (18), cT3–4 (35)	cN0(68), cN + (54)	0	21	9	0	23	0	0
H. S. Kim et al. [31]	6.5 (3.5–10.0)	Tis(3), cT1(7), cT2(3), cT3 (23), cT4(2)	cN0(18), cN + (20)	9 (23.1)	15 (9)	4 (4)	15 (14)	16	0	0
Marks et al. [25]	1.94 (3.11)	cT0 (7); cT1(4); cT2(9), cT3(31), cT4(2)	NR	41 (69.49)	8	22	0	12	30	0
Jung Cho et al. [28]	NR	NR	NR	NR	2	0	0	8	0	0
Suk Choi et al. [39]	NR	NR	NR	0	26	4	0	9	0	0

*a.v.* anal verge, *RC* right colectomy, *LC* left colectomy, *CME* complete mesocolic excision, *RAR* rectal anterior resection, *TME* total mesorectal excision, *TATA* transanal transabdominal radical proctosigmoidectomy, *NR* not reported, * mean ± SD

### Technical details of Da Vinci® Single-Port system

The technical details of DVSP are shown in Table 4. Nine studies [21–24, 26–30] reported the site of the single-port incision. Specifically, three studies utilized a Pfannenstiel incision [21, 22, 27], five employed a transumbilical incision [26–28, 30, 31], and five used an incision in the right lower quadrant [22, 23, 28, 29, 32]. The median length of the incision for Uniport placement was 4 cm (interquartile range (IQR) = 0.125 cm). All studies provided data on the utilization of additional ports. In nine of these studies [21–26, 28, 29, 31], an extra trocar was inserted.

**Table 4:** Technical details of Da Vinci SP

Author	Uniport position	Uniport type	Additional port
Song et al. [21]	Pfannenstiel incision	Dalim Biotech Ltd., Seoul, S. Korea	*n* = 5; 5-mm in the LLQ
Ha Lim et al. [27]	Trans‐umbilical incision (*n* = 40), Pfannesteil (1)	Custom‐made glove port	0
Piozzi et al. [22]	ISR (30 mm transverse incision at the RLQ); RC (30 mm Pfannenstiel incision)	Uni-port system (Daelim) or a Da Vinci SP ® Access Port	ISR (5 mm port in the RSQ); RC (A 12 mm port in the LLQ)
Alshalawi et al. [23]	RLQ	Dalim	*n* = 15; RSQt
Hye Jeong et al. [29]	RLQ	Uniport (Dalim)	*n* = 13; 10 cm cephalic to the SP trocar
Jin Kim et al. [32]	RLQ	Uniport (Dalim)	*n* = 42; in the RSQ
Jung et al. [26]	Transumbilical incision	Da Vinci SP ® Access Port	LC: 12 mm port in the RLQ; RC through a single port or additional 5 mm port in the LLQ
H. S. Kim et al. [31]	Transumbilical incision	Glove port + Uniport (Dalim)	*n* = 16
Marks et al. [25]	NR	mini GelSeal cap	3
Jung Cho et al. [28]	Transumbilical incision (*n* = 8)/right lower quadrant (n = 2)	SP glove port (Meditech Inframed, Seoul)	*n* = 1, in the RLQ
Suk Choi et al. [39]	Transumbilical incision	Handmade glove port	0

*ISR* intersphincteric resection, *RC* right colectomy, *LLQ* left lower quadrant, *RLQ* right lower quadrant, *RSQ* right superior quadrant, *mean ± SD, *NR* not reported

### Intraoperative outcomes

The intraoperative details of the patients are shown in Table 5. The median docking time was reported in 7 studies [21–23, 25, 28, 30, 31], with a median of 5.96 min (interquartile range (IQR), 9.33 min). The median console time was reported in 5 studies [21–23, 30, 31], with a median of 105 min (interquartile range (IQR), 62.51 min). The mean operative time (reported in all trials) was 186.3 min (interquartile range (IQR), 77.65 min). It is noteworthy that only the studies by Jung et al. [26] and Marks et al. [25] reported a single intraoperative complication (0.47%), with the latter specifically documenting a twisted anastomosis. Conversion to an alternative approach was documented in three cases (0.72%) [25, 26]. Marks et al. attributed the conversion to particularly challenging anatomy circumstances and suboptimal visualization, necessitating a transition from single-port to multiport laparoscopic surgery. The mean intraoperative blood loss was reported as 55 ml (median interquartile range (IQR) = 84.5 ml). A diverting ileostomy was constructed in 85 (20%) patients. Perioperative complications occurred in 52 patients (12.47%). These complications included paralytic ileus requiring a nasogastric tube in seven cases (13.46%), anastomotic leaks in five (9.61%), surgical site infections (SSI) in five (9.61%), bleeding (9.61%) in five, urinary retention in three (5.77%), voiding difficulties in two (3.84%), and chylous discharge occurred in one (1.92%) patient.

**Table 5:** Intraoperative details

Author	Port placing time, min	Docking time, min, median (range)	Console time, min, median (range)	Total operation time, min, median (range)		IOC *n*, (%)
Song et al. [21]	NR	4 min 40 s (4 min 10 s–5 min 10 s)	105 (100–120)	160 (150–240)		0
Ha Lim et al. [27]	NR	NR	NR	182 (121–305)		0
Piozzi et al. [22]	14 ± 5.11*	6.31 ± 2.92*	158 ± 46.69*	278.08 ± 84.79*		0
Alshalawi et al. [23]	NR	4 (2–10)	77.5 (46–99)	186 (117–225)		0
Hye Jeong et al. [29]	NR	NR	NR	180.0 ± 39.2*		0
Jin Kim et al. [32]	NR	NR	NR	160.0 (42.2)		0
Jung et al. [26]	NR	NR	NR	150.0 (130.0–201.0)		1
H. S. Kim et al. [31]	NR	14 (10–18)	200.0 (148.8–231.3)	262.5 (215.0–325.0)		0
Marks et al. [25]	NR	5.6 ± 4*	NR	357.15 (101.41)		1 (1.07)
Jung Cho et al. [28]	NR	less than 3 min	NR	222.4 ± 66.1 (142–316)		0
Suk Choi et al. [39]	NR	14.47 ± 10.38*	95.49 ± 35.33*	186.59 ± 51.3*		0
Incision size, cm, median	EBL, ml, median (range)	Drain	Diverting stoma, *n* (%)	Conversion, *n* (%)	Complications, *n* (%)	Clavien-Dindo
4	20 (10–20)	NR	0	0	1	II
4–6	100 (30–300)	NR	0	0	1	II
3	< 50	RC (not routine); ISR (always)	7	0	4	IIIa
4	20 (20–50)	15	3	0	1	II
4	20 (5–20)	13	7	0	1	IIIb
4.0 ± 0.3*	27.3 (38.5)	NR	16	0	5	II
5.0 (4.0–5.2)	10	NR	NR	1	4	II
NR	50 (45–100)	NR	4	0	5	II
4.5 (3.5–7)	50 (20–290)	8 (8.60)	46 (49.46)	2 (2.15)	14 (15.05)	IIIb
4	60.0 ± 38.7*	NR	NR	0	7	II
3.25 ± 0.6*	148 ± 126.2*	NR	2	0	1	II
3–4	103.33 ± 66.78*	NR	0	0	8	II

*IOC* intraoperative complications, *EBL* estimated blood loss, *LOS* length of stay, *ISR* intersphincteric resection, *RC* right colectomy, *NR* not reported, *mean ± SD

### Postoperative outcomes

The postoperative characteristics of the patients are shown in Table 6. Six papers [21, 22, 24, 25, 27, 31] reported data on the median time to the first flatus, which was 2.5 days (interquartile range (IQR) = 0.375). The median time to soft food was reported in five studies [21–24, 31], with a median of 2.5 days (interquartile range (IQR) = 0.9). The median length of hospital stay was 7.7 days (interquartile range (IQR) = 1.92). Five papers reported data on readmission [21, 23, 27, 28, 30], and no patient was readmitted for complications within 30 days.

**Table 6:** Postoperative details

Author	Time to first flatus, days (range)	Time to soft diet, day, median (range)	LOS, day, median (range)	Readmission (within 30 days), *n*
Song et al. [21]	2 (1–4)	5 (3–7)	7 (5–12)	0
Ha Lim et al. [27]	3 (1–4)	NR	6 (4–17)	0
Piozzi et al. [22]	1.82 ± 1.08*	2.5 ± 2.36*	6.46 ± 8.14*	NR
Alshalawi et al. [23]	NR	1.55	8 ± 4*	0
Hye Jeong et al. [29]	NR	NR	7.0 (6.0–8.0)	NR
Jin Kim et al. [32]	NR	NR	6.2 ± 1.7*	NR
Jung et al. [26]	NR	NR	5.0 (4.0–6.0)	NR
H. S. Kim et al. [31]	2.0 (2.0–3.0)	2.0 (2.0–2.0)	7.0 (7.0–8.0)	NR
Marks et al. [25]	2 (1–5)	NR	4 (1–24)	NR
Jung Cho et al. [28]	NR	NR	6 ± 1.2*	0
Suk Choi et al. [39]	NR	NR	7.95 ± 2.87*	0

*IOC* intraoperative complications, *EBL* estimated blood loss, *LOS* length of stay, *ISR* intersphincteric resection, *RC* right colectomy, *NR* not reported, *mean ± SD

### Oncological outcomes

The pathological data of the specimens are shown in Table 7. The median distance from the proximal resection margin (PRM) was 18.54 cm (interquartile range (IQR) = 38.02 cm), as reported by four papers [22, 24, 30, 31]. Seven studies [22–24, 27, 30–32] reported data on the median distance from the distal resection margin (DRM), which was 9.2 cm (interquartile range (IQR) = 4.24 cm). Six studies reported data on the circumferential resection margin (CRM) [24, 29–32], with only 2 out of 280 (0.71%) patients presenting a positive CRM. The median number of retrieved lymph nodes was 24.59 (interquartile range (IQR) = 12.4). The 30-day mortality rate was reported in seven trials [21, 23, 27–31], with no deaths recorded.

**Table 7:** Pathological details of the specimen

Author	Distance to PRM, cm, median (range)	Distance to DRM, cm, median (range)	CRM positive (< 1 mm), *n*	Resection margin involved, *n*	Retrieved lymph nodes, median (range)	Positive lymph nodes, median (patients, *n*)
Song et al. [21]	NR	NR	NR	NR	41 (39–50)	NR (3)
Ha Lim et al. [27]	NR	6.5 (1.5–20.5)	NR	NR	27 (12–79)	NR
Piozzi et al. [22]	26.1 ± 10.64*	8.04 ± 9.96*	NR	NR	23.67 ± 11.85*	0.5 ± 1.66*
Alshalawi et al.[23]	NR	3.52 (0.3–8.7)	NR	NR	16.5	NR
Hye Jeong et al. [29]	NR	NR	1	NR	26.0 ± 9.3*	NR (6)
Jin Kim et al. [32]	NR	2.2 ± 1.7*	0	NR	17.8 (9.9)	NR (15)
Jung et al. [26]	NR	NR	NR	NR	NR	NR
H. S. Kim et al. [31]	120 (75–160)	47.0 (20–71)	0	NR	18 (12.0–20.0)	0 (0.0–2.0)
Marks et al. [25]	NR	NR	0	0	24 (11–58)	1 (0–1)
Jung Cho et al. [28]	NR	NR	NR	NR	NR	NR
Suk Choi et al. [39]	10.97 ± 7.62*	10.02 ± 10.15	1	NR	24.59 ± 12.82*	NR
Author	Postoperative pathological stage, (*n*)		Adjuvant chemotherapy, *n* (%)		Mortality in the first post 30-days, *n*	Follow up, months, mean (SD)
Song et al. [21]	I (1), IIA (1), IIIB (3)		3		0	NR
Ha Lim et al. [27]	0/I (13), II (9), III (16), IV (3)		NR		0	NR
Piozzi et al. [22]	pT0 (2); pT1 (4); pT2 (1); pT3 (5); pN0 (11); pN2a (1)		5		NR	8 (3.74)
Alshalawi et al.[23]	NR		NR		0	NR
Hye Jeong et al. [29]	yTx (6), yT1 (2), yT2 (4), yT3 (3), yT4 (5), yN0 (14), yN + (6)		8		0	NR
Jin Kim et al. [32]	pTx (5)/T1 (5)/T2(10)/T3 (22); pN0 (27), N1 (12) N2(3)		30		0	NR
Jung et al. [26]	0–II (33), III–IV(20)		NR		NR	NR
H. S. Kim et al. [31]	Pathologic Stage 0 (5); 1 (8); II (6), III (19), IV (0)		NR		NR	3
Marks et al. [25]	pT0 (14); pT1 (3); pT2 (14); pT3 (10); pT4 (1)		13 (30.23)		NR	23 (6)
Jung Cho et al. [28]	0/I (6), IIA (2), IIIA (1), IIIB (1)		4		0	4.3 (1.7)
Suk Choi et al. [39]	I (14), II (6), III (18),IV(1)		0		0	NR

*PRM* proximal resection margin, *DRM* distal resection margin, *mean ± SD, *NR* not reported

## Discussion

This study is the inaugural comprehensive systematic review to rigorously evaluate the feasibility and effectiveness of the Da Vinci SP robotic platform (DVSP) in colorectal surgery. Our findings suggest that DVSP seems to be a viable and safe approach, with successful procedure completion and with encouraging surrogate oncological parameters.

The DVSP system signifies a substantial advancement in robotic surgery, offering notable enhancements over earlier multiport robotic systems. Notably, while it has not yet received FDA approval for general surgery in the USA  and is currently restricted to experimental protocols, the system has been utilized in a wide range of surgical procedures, encompassing outpatient surgery and complex oncological interventions, including hepatobiliary and rectal surgery. It has recently received the European CE marking, and several groups across Europe have begun to introduce it into clinical practice.

The existing data on this platform primarily focus on urological procedures [33] and gynecology. In urology, the system has been successfully utilized for procedures such as radical prostatectomy, with studies highlighting its feasibility, safety, and benefits including reduced postoperative pain and shorter hospital stays [14, 34]. Similarly, in gynecology, the DVSP has been employed for hysterectomy, myomectomy, and oncologic surgeries with promising outcomes such as minimal blood loss, reduced complications, and faster recovery times [35–37]. These applications underscore the platform’s ability to enhance surgical precision while minimizing invasiveness. The extensive experience in these fields provides valuable insights into the safety and feasibility of single-port robotic surgery, which can inform its expanding use in colorectal procedures.

## Intraoperative outcomes

The platform was used in 417 colorectal procedures, resulting in a low conversion rate (0.72%). Only two intraoperative (0.47%) and 52 postoperative complications (12.47%) were reported. The complication rates observed in this study are consistent with those reported in the existing literature [32, 38].

The technical specifications of the DVSP platform are notable for its median docking time of 5.96 min (IQR, 9.33 min), which is a significant advantage over competing platforms. The docking process is intuitively designed, readily mastered, and expeditious when compared to multiport robotic platforms. Two studies by Choi et al. [30, 39], published in 2022 and 2024, assessed the learning curve in colorectal DVSP surgery, focusing on procedure and docking times as key metrics. The authors found that optimal stabilization was achieved after 18 colectomy cases and 21 rectal cancer surgeries, with mean docking times of 14.87 ± 10.38 min and 20.6 ± 19.1 min, respectively.

In comparison, Marks et al. [25] reported that process control for docking in abdominal DVSP cases was achieved after 45 cases, with a mean docking time of 5.60 min. These docking times are shorter than those previously reported with the multiport da Vinci Xi or Si robotic platforms [40]. However, it is important to note that the DVSP platform does have certain limitations. The absence of integrated staplers and energy instruments necessitates the presence of a highly skilled assistant at the operating table and potentially additional trocars to compensate for these deficiencies. However, advances in tools like energy devices, suction-irrigation systems, and staplers could reduce the reliance on a bedside assistant and support true single-site surgeries, eliminating the need for additional trocars [41]. The median total operative time was 186.3 min interquartile range (IQR), 77.65 min). A paucity of comparative studies exists between DVSP and multiport robotic or laparoscopic colorectal surgery. A propensity score-matched analysis was conducted by Jung et al. [26] to compare the Da Vinci SP and Xi systems, finding no significant difference in operative times. In contrast, a retrospective study by Jin Kim et al. [24] reported a significantly shorter operative time for robotic TME using the DVSP system. However, in a comparison between multiport laparoscopy and DVSP, Seung Kim et al. [31] showed that the former had significantly shorter operative times.

With respect to intraoperative characteristics, the rate of intraoperative complications was remarkably low, with only two complications reported in the entire cohort (0.47%), including one case of twisted anastomosis. Postoperative complications were more extensively documented, with 52 cases (12.47%) reported. Of these, ileus was the most prevalent, accounting for 13.46% of all post-operative complications.

## Postoperative outcomes

A primary objective of DVSP is to enhance the aesthetic outcomes of surgical interventions [42]. The median length of the incision for Uniport placement was 4 cm (interquartile range (IQR) = 0.125 cm). Bianco et al. evaluated cosmetic outcomes following 222 procedures using the DVSP and reported a mean satisfaction score of 9.2 (range 4–10) after approximately 9 months of follow-up [43]. A notable limitation of this technology pertains to the augmented risk of incisional hernia formation, attributable to the necessity of a more substantial incision for the 2.5 cm port. However, a comparative analysis of incisional hernia rates between multi-port (1.1%) and single-port (1.3%) robotic TAPP procedures after 13 months of follow-up revealed no significant difference, as reported by Bianco et al. [44].

The median length of hospital stay was 7.7 days (interquartile range (IQR) = 1.92). Concurrently, Min Hye Jeong et al., Hye Jin Kim et al., and Jin-Min Jung et al. reported a shorter LOS for DVSP TME patients in comparison to those undergoing multiport robotic TME [24, 26, 29]. Consistent findings were reported by Jeong et al. and Kim et al. in their DVSP series, where both LOS and estimated blood loss (EBL) were significantly lower compared to multiport (MP) procedures [29, 32]. In contrast, Ha Lim et al. [27] compared single-port robotic and single-port laparoscopic right colectomy and found no significant differences in LOS between the two approaches.

## Perspectives

A salient finding of this analysis was the successful oncological precision achieved with the DVSP, as evidenced by a 99.29% rate of R0 resection, although this data was reported in only two studies [25, 32]. This high success rate underscores the system’s effectiveness in ensuring complete tumor removal, a critical factor in minimizing recurrence and enhancing long-term survival for colorectal cancer patients.

Additionally, the median retrieval of 24.59 lymph nodes (interquartile range (IQR) = 12.4) aligns with established oncological standards for adequate cancer staging and resection, further substantiating the platform’s aptitude for advanced oncological procedures.

## Unanswered questions, underexplored areas, and limitations

This study has several limitations, including the relatively small number of reported cases and the majority of the included studies conducted in Korea, the lack of long-term follow-up, unavailability of recurrence rates and overall patient prognosis, and variability in techniques used. The learning curve and its impact on surgical outcome for DVSP in comparison to other platforms is yet to be elucidated. Although retrieved lymph nodes appear acceptable, the absence of survival data and limited information on nodal positivity precludes definitive conclusions regarding short-term oncological outcomes. Widespread adoption and future randomized trials with robust long-term follow-up are essential for a more thorough evaluation of surgical and oncological outcomes.

The widespread adoption of robotic systems in colorectal surgery faces several challenges, particularly its high costs, with initial investments ranging from $1 to $2.5 million and substantial maintenance expenses [45, 46]. Given the financial burden and the lack of demonstrated superiority, further studies are essential to determine the true value of the DVSP in colorectal surgery.

## Conclusion

This study shows that the use of DVSP in colorectal surgery is both feasible and safe. The preliminary findings, particularly those pertaining to short-term results and surrogate oncological outcomes, are encouraging. However, the current literature is limited by the need for long-term follow-up data and larger patient cohorts. These elements are crucial for validating the functional and oncological outcomes associated with this platform and for effective comparison with other established minimally invasive techniques.

## Supplementary Information

Below is the link to the electronic supplementary material.

Supplementary file1 (PDF 195 KB)

Supplementary file2 (PDF 79 KB)

Supplementary file3 (DOCX 63 KB)

## Data Availability

The data that support the findings of this study are available on request from the corresponding author, [F.B.].
