# Supplementary material for: A systematic review of the Da Vinci® Single-Port system (DVSP) in the context of colorectal surgery
Source: Int J Colorectal Dis. 2025 Apr 2;40(1):83. doi: 10.1007/s00384-025-04878-x (PMC11965226; doi:10.1007/s00384-025-04878-x)

**Detailed search strategy**

**Pubmed**

("Da Vinci SP" OR "single-port" OR "single-site" OR "single-incision" OR "SILS") AND ("Robotic Surgical Procedures"[MeSH] OR "robotic" OR "robot-assisted") AND ("Colorectal Surgery"[MeSH] OR "Colorectal Neoplasms"[MeSH] OR "Colectomy"[MeSH] OR "Proctectomy"[MeSH] OR "colorectal" OR "colon" OR "rectal" OR "colectomy" OR "proctectomy")

**Cochrane library**

("da Vinci SP" OR "single-port" OR "single-site" OR "single-incision" OR SILS)

AND

("robotic" OR "robot-assisted" OR "robotic surgery")

AND

("colorectal" OR "colorectal neoplasm*" OR "colectomy" OR "proctectomy" OR "colon" OR "rectal")

**Web of Science**

TS=("da Vinci SP" OR "single-port" OR "single-site" OR "single-incision" OR SILS)

AND

TS=("robotic surgery" OR "robotic" OR "robot-assisted" OR "robot-assisted surgery" OR "robotic system*")

AND

TS=("colorectal surgery" OR "colorectal neoplasm*" OR "colon cancer" OR "rectal cancer" OR colorectal OR colon OR rectal OR colectomy OR proctectomy)

657 total results

**Fig. 1s Detailed Risk of Bias assessment**


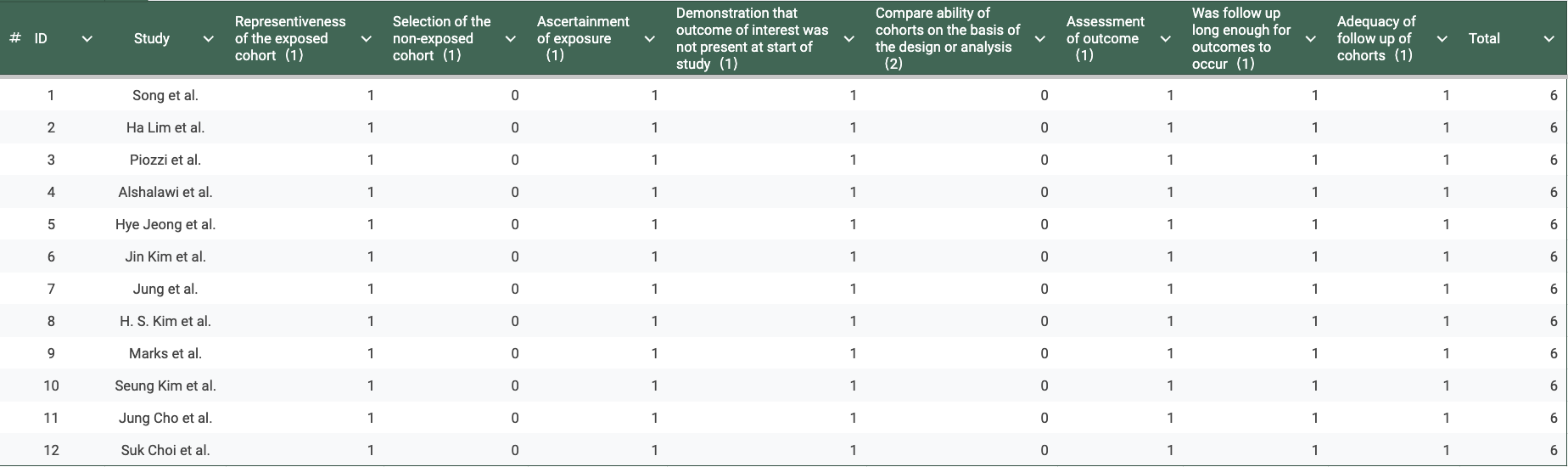

Supplement: Supplementary file 3 — Supplementary file3 (DOCX 63 KB) [file 384_2025_4878_MOESM3_ESM.docx]
